# Gene expression profiling in murine autoimmune arthritis during the initiation and progression of joint inflammation

**DOI:** 10.1186/ar1472

**Published:** 2004-12-14

**Authors:** Vyacheslav A Adarichev, Csaba Vermes, Anita Hanyecz, Katalin Mikecz, Eric G Bremer, Tibor T Glant

**Affiliations:** 1 Section of Biochemistry and Molecular Biology, Department of Orthopedic Surgery, Rush University Medical Center, Chicago, Illinois, USA; 2 Children's Memorial Institute for Education and Research, Northwestern University, Chicago, Illinois, USA

**Keywords:** DNA expression array, differential gene expression, inflammation, arthritis-related genes, rheumatoid arthritis

## Abstract

We present here an extensive study of differential gene expression in the initiation, acute and chronic phases of murine autoimmune arthritis with the use of high-density oligonucleotide arrays interrogating the entire mouse genome. Arthritis was induced in severe combined immunodeficient mice by using adoptive transfer of lymphocytes from proteoglycan-immunized arthritic BALB/c mice. In this unique system only proteoglycan-specific lymphocytes are transferred from arthritic mice into syngeneic immunodeficient recipients that lack adaptive immunity but have intact innate immunity on an identical (BALB/c) genetic background.

Differential gene expression in response to donor lymphocytes that migrated into the joint can therefore be monitored in a precisely timed manner, even before the onset of inflammation. The initiation phase of adoptively transferred disease (several days before the onset of joint swelling) was characterized by differential expression of 37 genes, mostly related to chemokines, interferon-γ and tumor necrosis factor-α signaling, and T cell functions. These were designated early arthritis 'signature' genes because they could distinguish between the naive and the pre-arthritic state. Acute joint inflammation was characterized by at least twofold overexpression of 256 genes and the downregulation of 21 genes, whereas in chronic arthritis a total of 418 genes with an equal proportion of upregulated and downregulated transcripts were expressed differentially.

Hierarchical clustering and functional classification of inflammation-related and arthritis-related genes indicated that the most common biological activities were represented by genes encoding interleukins, chemokine receptors and ligands, and by those involved in antigen recognition and processing.

## Introduction

The completion of the human and mouse genome sequencing programs and the subsequent annotation of previously unidentified genes have opened a new epoch in biology and biomedical sciences. The genetic information greatly facilitated the discovery of novel disease-related genes and the mapping of signature genes for early diagnosis. More specifically, polynucleotide or oligonucleotide arrays have been applied in both human and experimentally induced disease conditions to determine characteristic expression patterns of signature genes.

In an inflammatory disease such as rheumatoid arthritis (RA), the gene expression profile is extremely complex owing to the diversity of cell types involved in the pathology and the polygenic character of the autoimmune disease [1-5]. The overall picture of molecular interactions in an inflamed joint, deduced from gene expression studies in both RA and its corresponding animal models, involves proteins participating in immunity, inflammation, apoptosis, proliferation, cellular transformation and cell differentiation, and other processes [3-8]. Several studies analyzed the patterns of gene expression in peripheral blood or synovial fluid mononuclear cells, and in the inflamed synovium of human patients [1,3-5,7,9-11]. However, the genetic heterogeneity of the human population is a serious obstacle to the correct interpretation of data in gene expression studies. Animal models of RA can facilitate the interpretation of genome-wide gene expression by providing genetic and clinical homogeneity, and an opportunity to monitor the onset and progression of the disease [12-20]. DNA microarray technology was successfully applied to inflamed paws of mice or rats systemically immunized with arthritogenic compounds to induce arthritis [6,21-23]. Despite the usefulness of the information provided by these studies, the early gene expression events at the site of inflammation (joint and synovium) and the mechanisms of disease initiation remain unknown.

Systemic immunization of genetically susceptible BALB/c mice with human cartilage proteoglycan aggrecan (PG) induces PG-specific immune responses that then trigger inflammation in peripheral joints [13,19]. PG-induced arthritis (PGIA) is a murine model which bears many similarities to RA as indicated by clinical assessments, radiographic analyses, various laboratory and functional tests, and by histopathologic studies of diarthrodial joints [13,19,24,25]. Moreover, genome-wide screening studies identified multiple genomic loci in PGIA [20,26-29] that are syntenic with those described in RA [25]. Both RA and PGIA are polygenic autoimmune diseases with a major permissive role of the MHC, although non-MHC genes account for a significant portion of the genetic susceptibility. PGIA can be successfully transferred into naive BALB/c or syngeneic severe combined immunodeficient (SCID) mice either with unseparated spleen cells or with antigen (PG)-stimulated T lymphocytes from arthritic donor BALB/c mice [30-32].

In the present study, we adoptively transferred the disease (PGIA) into syngeneic BALB/c^SCID ^mice lacking functional T and B cells. SCID mice carry a natural mutation that prevents the V(D)J recombination in B and T lymphocytes, resulting in a failure to generate functional immunoglobulins and T cell receptors [33,34]. Consequently, adoptively transferred arthritis in BALB/c^SCID ^mice is an ideal model in which activated lymphocytes of arthritic donor BALB/c mice migrate and interact with the intact innate immunity environment in the joints of BALB/c^SCID ^mice. The gene expression profiles in normal, pre-arthritic and arthritic joints of the recipient BALB/c^SCID ^mice were determined by using DNA microarray technology (Affymetrix). Although a significant number of genes were differentially expressed in joints with acute and chronic arthritis, in this study we focused on early genes whose expression occurred before the onset of clinical symptoms.

## Methods

### Animals, antigen and immunization

The use of human cartilage from joint replacement surgeries for antigen isolation was approved by the Institutional Review Board, and all animal experiments were approved by the Institutional Animal Care and Use Committee. Female BALB/c mice at the age of 24–26 weeks (National Cancer Institute, Kingston Colony, New York, USA) were injected intraperitoneally with 100 μg of cartilage PG (measured as protein) emulsified in dimethyldioctadecylammonium bromide (DDA) adjuvant (Sigma-Aldrich, St Louis, Missouri, USA). The use of adjuvant DDA allowed us to avoid the harmful effects of oil and bacterial proteins present in Freund's adjuvants [35,36]. Booster injections of the same doses of PG with DDA were given on days 21 and 42. BALB/c mice develop swelling and redness of one or more limbs 7–10 days after the second or third injection with PG in adjuvant [25]. Arthritis was assessed daily, and inflammation was scored from grade 0 to grade 4 for each paw [13,36,37]. Female SCID mice of the BALB/c background (NCI/NCrC.B-17-scid/scid; henceforth BALB/c^SCID^) were used for adoptive cell transfer. BALB/c^SCID ^mice were purchased from the National Cancer Institute and maintained under germ-free conditions.

### Stimulation of lymphocytes *in vitro*, and adoptive transfer of arthritis

To ensure uniformity and reproducibility of disease transfer, donor spleen cells were isolated from arthritic BALB/c mice within 1–2 weeks after the onset of inflammation. At least two paws of donor BALB/c mice were arthritic, and the cumulative inflammation score (for four paws) was in the range 5–8. Spleen cells of arthritic BALB/c mice were collected and cultured in six-well plates (2.5 × 10^6 ^cells/ml) with cartilage PG (50 μg/ml) for 4 days in Dulbecco's modified Eagle's medium supplemented with 5% fetal bovine serum (HyClone Laboratories, Logan, Utah, USA). After stimulation *in vitro *for 4 days with cartilage PG, non-adherent cells were collected, and live cells (lymphocytes) were separated on Lympholyte-M (Cedarlane, Ontario, Canada). Finally, 2 × 10^7 ^lymphocytes were injected intraperitoneally on days 0 and 7 into recipient BALB/c^SCID ^mice as described [32].

A standard scoring system used for primary arthritis was applied to the assessment of disease severity in BALB/c^SCID ^mice [24,37]. Typically, one to four paws became inflamed simultaneously 3–5 days after the second cell transfer, and the rest of the peripheral joints became inflamed within 2–4 days after the onset of the first symptoms. BALB/c^SCID ^mice were scored twice daily, and were killed as soon as the inflamed paw reached an individual arthritis score of 2, but not later than 24 hours after the onset of arthritis. This paw was designated as acute arthritic (AA), and contralateral or ipsilateral paws that were not inflamed at that time were used as pre-arthritic (PA) samples. The PA joints did not show evidence of inflammation on histopathological examination, although thickening of the synovial lining in small joints was observed occasionally (data not shown). Several arthritic BALB/c^SCID ^mice were scored daily and were killed 8–10 days after disease onset. These joint samples represented subacute-chronically arthritic (CA) samples. In addition to PA, AA and CA experimental conditions, paws of naive non-immunized BALB/c^SCID ^mice were used as 'absolutely negative' (control naive; AN) samples for RNA isolation and subsequent hybridization. Each sample represented RNA pooled from four paws of two mice.

### Probe preparation

Synthesis and biotinylation of cRNA and hybridization were performed in accordance with the manufacturer's instructions (Affymetrix, Santa Clara, California, USA). In brief, total RNA was isolated from normal or inflamed paws of mice by using TRIzol reagent (Invitrogen, Gaithersburg, Maryland, USA) with additional purification on RNeasy columns (Qiagen, Valencia, California, USA). RNA quality was confirmed by spectrophotometry and electrophoresis on formaldehyde gels [38]. Double-stranded complementary DNA was synthesized with the T7-dT24 primer incorporating a T7 RNA polymerase promoter. Biotinylated cRNA was prepared with the Enzo BioArray High Yield RNA Transcript Labeling Kit (Enzo Diagnostics, Inc., Farmingdale, New York, USA) and hybridized to the murine genome Affymetrix U74v2 chip set, which included three DNA chips, MG_U74Av2, MG_U74Bv2 and MG_U74Cv2, interrogating more than 36,000 genes that represented essentially the entire mouse genome [39-42]. Fluorescent hybridization signals were developed with phycoerythrin-conjugated streptavidin and were further enhanced with fluorescently labeled anti-streptavidin antibodies. DNA chips were scanned to obtain quantitative gene expression levels. DNA chip hybridization, Fluidics Station operations, scanning, and preliminary data management were performed in accordance with Affymetrix protocols as described previously [43,44].

### Microarray analysis

Fluorescent intensity data from Affymetrix Microarray Suite version 5 were exported as CEL files and imported into DNA-Chip Analyzer version 1.3 [45]. Data were normalized, and expression values, based on the perfect match/mismatch (PM/MM) model, were calculated for each DNA chip. All chips were examined for the image spikes, chip and gene outliers. Exported expression values for each DNA chip were combined into a single file (three chips × four experimental conditions × three to five replicates), and imported back to DNA-Chip Analyzer; the resulting data were normalized by using an array with median probe intensity.

For the pairwise comparison of experimental conditions, signals were filtered by using several criteria. Gene expression was considered above the background if it showed the signal on most chips (more than 50%; that is, for three replicates, the gene should be detectable on at least two chips; for five replicates, the gene should be present on at least three DNA chips). Fold changes for gene expression were calculated when any of three following criteria were met: (1) the gene was present in the experimental condition but absent in the basal condition; (2) the gene was present in the basal condition but absent in the experimental condition; (3) the gene was present in both basal condition and experimental conditions. Student's *t*-test was used to determine the statistical significance of the difference in gene expression between basal and experimental conditions (*P *< 0.05 was taken as significant). An additional cut-off threshold of twofold change in gene expression (either upregulation or downregulation) was used to characterize a gene as being differentially regulated (for example, a negative twofold value corresponded to a twofold downregulation). The Fisher exact test (implemented by us in Visual Basic code for MS Excel 2000) and the Mann–Whitney *U*-test (SPSS, Chicago, Illinois, USA) were used to verify non-paired Student's *t*-test calculations of the probability of gene expression differences in pairwise comparisons. Finally, the false discovery rate was established with 500 permutations for each pairwise comparison to estimate the proportion of false-positive genes.

To characterize gene expression patterns, hierarchical gene clustering was performed with a DNA-Chip Analyzer program [45,46]. The algorithm was based on the distance between two genes defined as 1 - *r*, where *r *is the Pearson correlation coefficient between the standardized expression values of the two genes across the samples used. To characterize functional relationships between differentially expressed genes, Gene Ontology terms classification [47], incorporated in DNA-Chip Analyzer, was performed [48]. The significance level for a functional cluster was set at *P *< 0.05, and the minimum size of a cluster was three genes. Venn diagram calculations were performed in Visual Basic code for MS Excel 2000 to analyze overlapping of sets of genes differentially expressed in the samples at different phases of arthritis.

## Results

The major goal of the present study was to find and characterize early signature genes whose expressions were different (at least twofold change in the threshold level) and statistically significant (*P *< 0.05) between experimental groups at different phases of joint inflammation. The induction of arthritis in BALB/c^SCID ^mice was a multi-step process. First, donor BALB/c mice were immunized with cartilage PG to induce arthritis. Second, spleen cells from acutely arthritic (AA) donor mice were stimulated *in vitro *with cartilage PG, and live lymphocytes were isolated on a Lympholyte-M density gradient. Third, these antigen-stimulated donor lymphocytes were injected into BALB/c^SCID ^mice. For gene expression profiling during the time course of the adoptively transferred arthritis, RNA was isolated from pre-arthritic paws (PA) and diseased paws (AA and CA) (Table 1). In addition, RNA was isolated from normal paws of naive BALB/c^SCID ^mice and served as a baseline non-arthritic control condition (AN). Three pairwise comparisons were performed: PA versus AN, AA versus AN, and CA versus AN (hereafter denoted as PA/AN, AA/AN and CA/AN).

Each experimental condition was reproduced three to five times (RNA isolation, probe preparation, and independent hybridizations), and each replicate contained RNA samples pooled from a total of four paws of two arthritic animals. When the number of replicates is low and the distribution of data in the general population is basically unknown, the applicability of Student's *t*-test is questionable. We therefore analyzed data by using both Student's *t*-test and the Fisher exact test, in which the first approach requires normal data distribution, whereas the second test does not have this requirement [45,49,50]. Setting the significance level for the difference between groups at *P *< 0.05 and no threshold for the fold change in expression, 1805 genes passed the Fisher exact test and 1752 genes passed the DNA-Chip Analyzer Student's *t*-test [45] for the PA/AN comparison. In AA/AN pairwise comparisons, 3676 genes passed the Fisher exact test and 3305 genes passed Student's *t*-test. Concluding that Student's *t*-test provided similar results and was even more conservative than the Fisher exact test, we employed the former for all further analyses.

### Effect of the numbers of replicates on data variability

**Figure 1:**
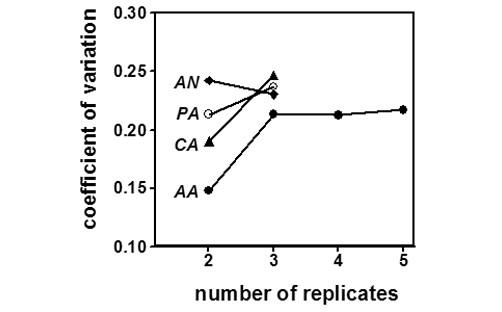
Average coefficient of variation with increasing number of replicates of gene expression experiments. Data represent results obtained with RNA from normal paws of naive BALB/c^SCID ^mice (AN), clinically normal pre-arthritic paws (PA), acutely arthritic paws (AA) and chronically inflamed paws (CA).

Being aware of the importance of data reproducibility, we determined the optimal number of arrays to be included in experimental design by monitoring the convergence of variance for gene expression signals in five replicates representing the condition AA. For each replicate, we pooled equal amounts of quality-controlled RNA samples, isolated from two inflamed paws of two BALB/c^SCID ^mice that had been identically treated (in terms of the number of donor cells and antigen stimulation) and had similar disease onset and severity. A total of five replicates represented 20 paws of 10 arthritic mice. We used the coefficient of variation (CV) to measure data variability. The CV for each gene on the chip and the mean CV for the entire probe set were calculated. Mean CV reached a plateau when the number of replicates increased beyond three (Fig. 1, experimental condition AA) and there was no significant change afterwards. Therefore, for all other experimental conditions, we used three replicates representing three independent hybridization experiments of three RNA samples isolated from six paws. Mean CV after sampling of the three repeats ranged between 0.21 and 0.25 for all experimental conditions.

### Arthritis 'signature' genes in pre-inflamed joints

**Figure 2:**
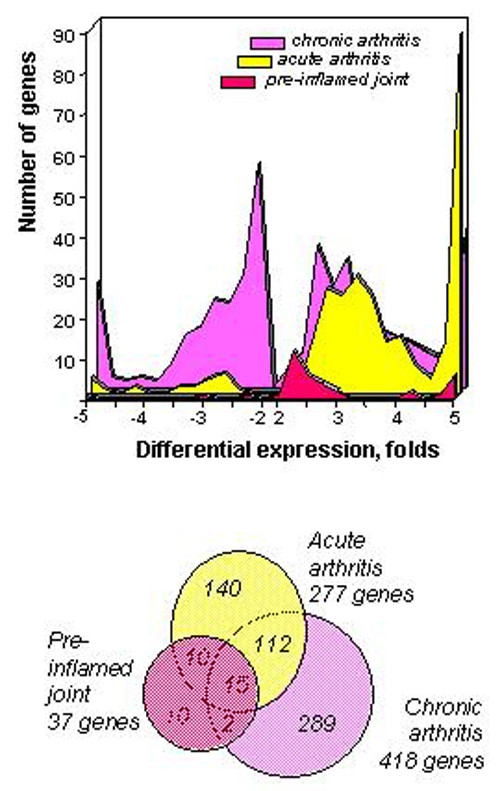
Fold change distribution for genes differentially expressed in pre-inflamed joints, in paws with acute and chronic arthritis, in comparison with gene expression in normal paws of naive BALB/c^SCID ^mice. Values indicate the number of genes that fall in the given range of expression. Negative numbers for expression levels indicate downregulation (e.g. a negative twofold change corresponds to downregulation to 0.5-fold). Spikes at ± 5-fold expression change represent the extremes of histogram when combining all genes with differential expression level greater than ± 5-fold. The Venn diagram (bottom) indicates the number of overlapping genes that were differentially expressed in pre-inflamed and arthritic joints.

Paws of naive BALB/c^SCID ^mice and still non-inflamed (PA) paws were clinically normal with no sign of inflammation, and comparison of these two experimental conditions (PA/AN) identified a relatively small number of differentially expressed genes. Only 37 of the 36,000 screened genes were differentially expressed (that is, showed greater than a ± twofold change relative to threshold level), of which 11 genes were over the ± threefold threshold, and seven genes changed beyond ± fivefold (Fig. 2). The seven genes with the most significant change in expression levels encoded chemokine CC motif receptor 5 (*Ccr5*), chemokine CXC motif ligand 1 (*Cxcl1*), interferon-γ-inducible protein (*Ifi47*), membrane-spanning 4-domains subfamily A member 6C (*Ms4a6c*), tumor necrosis factor-α-induced protein 6 (*Tnfip6*), T cell receptor β variable 13 (*Tcrbv13*), and Terf1-interacting nuclear factor2 (*Tinf2*) (Table 2). Although the upregulation of *Tcrbv13*, *Tgtp *and interferon-induced genes might indicate the appearance of antigen-specific T cells in the synovium (Table 2), the significant upregulation of *Tnfip6 *suggests the activation of an anti-inflammatory cascade [51]. Thus, gene expression related to pro-inflammatory and anti-inflammatory events can be detected even before the migration of inflammatory leukocytes into the joints.

**Figure 3:**
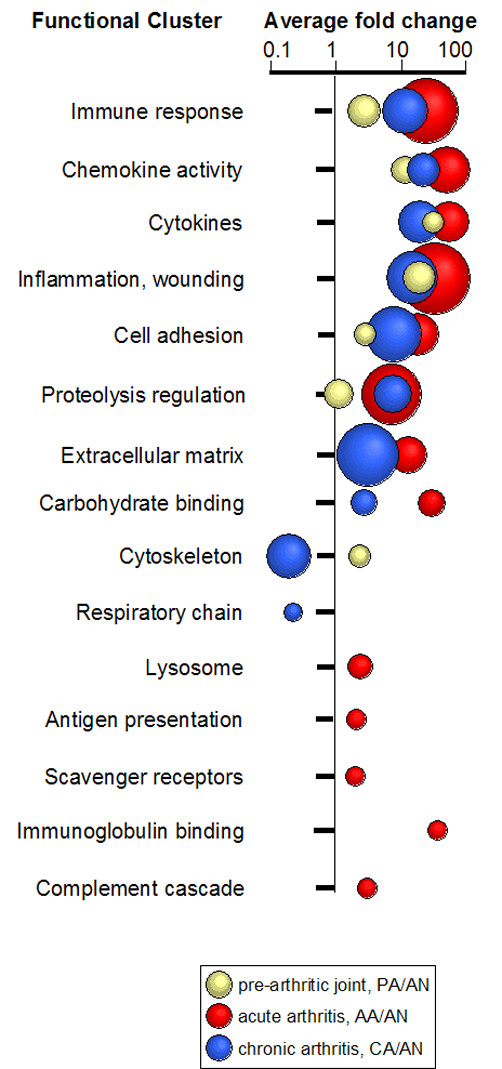
Gene activities at different phases of arthritis progression. All clusters identified in pre-inflamed joints (PA/AN comparison, yellow circles), acute arthritis (AA/AN, red circles), and chronic arthritic paws (CA/AN, blue circles) are indicated by the number of genes in the cluster (circle diameter represents cluster size) and the average fold change of gene expression (logarithmic horizontal scale). The size of the cluster varies from 3 genes ('complement cascade' cluster) in pre-inflamed joints to 51 genes ('immune response' cluster) in acute arthritis. AN, normal paws of naive BALB/c^SCID ^mice; PA, clinically normal pre-arthritic paws; AA, acutely arthritic paws; CA, chronically inflamed paws.

To characterize major biological functions in context with the initiation phase of the disease, we assigned the 37 early genes (Table 2, Additional file 1) to separate groups according to the corresponding protein functions and Gene Ontology classification [47,48]. We found that differentially expressed genes in PA joints were related to immune responses, chemokine activity (including chemotaxis), cell adhesion, proteolysis regulation, inflammation and wounding, cytokines, and cytoskeletal activity (Fig. 3, yellow circles). All clustered genes were upregulated at the pre-inflamed phase of arthritis.

### Gene expression profile in acute and chronic arthritis

To monitor the progression of disease, we analyzed genes that were differentially expressed in paws with acute and chronic joint inflammation. Both AA and CA experimental conditions were associated with the activity of a large number of genes: 256 genes were upregulated and 21 were downregulated in acute arthritis (AA/AN comparison), and 201 genes were upregulated and 217 were downregulated in chronic inflammation (CA/AN) (Fig. 2, Additional files 2 and 3). A Venn diagram summarizes the relationships between gene sets that were differentially expressed at different phases of the disease. Only 15 genes were differentially expressed in all three phases of the disease (PA, AA, and CA), 25 genes were differentially expressed both at the PA phase and during acute inflammation, 127 genes were active both in acute and chronic phases, and 17 transcripts shared a common expression pattern in pre-inflamed and chronically inflamed joints (Fig. 2).

Using Gene Ontology terms for the functional classification of genes differentially expressed in acute and chronic arthritis [47], dozens of cell signaling pathways and gene clusters were identified. By further filtering of functional clusters, and by combining clusters encoding proteins with similar functions, we found that the acute and chronic phases of the disease can be comprehensively described by the differential expression of 15 macro-clusters (Fig. 3). Six clusters were found in all three phases of inflammation; they were related to immune response, chemokine activity, cytokines, inflammation and wounding, cell adhesion, and proteolysis regulation. The most abundantly represented genes in inflamed joints were those involved in immune responses: 51 genes in AA and 25 genes in CA. These genes were upregulated as much as 31-fold (group average) in acute arthritis and 15-fold in chronic arthritis (Fig. 3). Cytokine and chemokine genes demonstrated the highest overexpression levels: about 64-fold in acute and 28-fold in chronic arthritis, where both groups included more than a dozen genes. Proteolysis-regulating genes (proteases and their inhibitors) were highly represented at the acute phase (45 genes), but were less abundant in chronic arthritis (19 genes). Extracellular matrix-related genes, mostly relevant to tissue repair and healing, were more abundant in chronic than acute disease. Some functional clusters were phase-specific, such as lysosome, antigen presentation, scavenger receptors, immunoglobulin binding, and complement cascade; these genes were preferentially expressed in acute joint inflammation. Suppression of genes related to the respiratory chain complex was specific to chronic inflammation (Fig. 3).

### Hierarchical clustering of arthritis phase-specific genes

**Figure 4:**
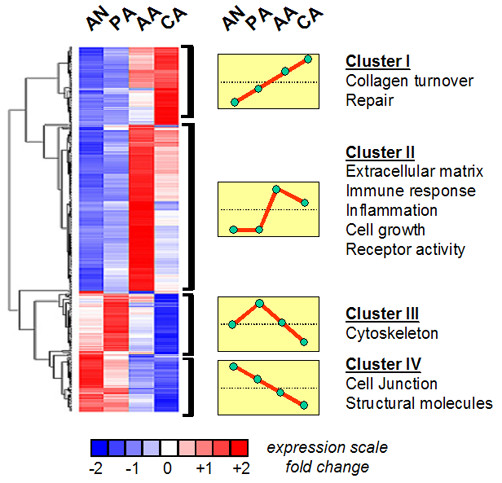
Signature gene clusters at different phases of autoimmune arthritis. Hierarchical clustering was performed for genes whose expression significantly differed when paws of naive mice (AN) were compared with those in the pre-arthritic (PA), acute (AA), or chronic (CA) phases of arthritis. The total number of genes (*n *= 507) is less than the sum of the phase-specific genes because of partial overlap (Fig. 2). Rows represent individual genes; columns represent individual expression values for each gene at the indicated phase of arthritis. The major biological activities, specific for each cluster, were examined by using functional clustering of genes. This analysis yielded four different expression patterns (clusters I–IV). Upregulated genes are shown in red, downregulated genes in blue.

To identify genes whose expression might be specific for the actual phase of arthritis, and to combine transcripts by the pattern of their expression through all disease phases, we applied a hierarchical clustering technique [46]. Genes that were specific for pairwise comparisons (PA/AN, AA/AN, and CA/AN) were combined into one single file (excluding redundant genes); the merged set included 507 genes. Hierarchical clustering was performed for all experimental conditions studied (AN, PA, AA, and CA), and four major gene clusters were identified, each with a distinct expression pattern (Fig. 4, clusters I–IV). Using further classification analysis with Gene Ontology terms, to examine the functions of genes inside each cluster, we identified genes encoding proteins whose biological functions were the most relevant to arthritis development and progression.

Cluster I contained genes with major functions in collagen turnover and tissue repair; the expression of these genes reached a peak in chronically inflamed joints.

Cluster II was the largest cluster including about half of all phase-specific genes (Fig. 4). The cluster included genes with roles in immune, inflammatory and stress responses, extracellular matrix formation, cell growth, and receptor activity. The expression of cluster II genes reached a peak at the acute phase of joint inflammation.

Transcription of genes in clusters III and IV gradually decreased during disease progression (Fig. 4). These genes were mostly related to cytoskeleton remodeling, the formation of cell junctions, and the production of structural molecules such as desmin, β-3 laminin, envoplakin, and dystonin (for a detailed gene list see Additional files 1, 2, 3). Genes associated with early arthritis (Table 2) were found in clusters III and IV, further underlining the importance of cell adhesion and cytoskeleton remodeling during the initiation phase of arthritis.

### Expression patterns of early arthritis genes

**Figure 5:**
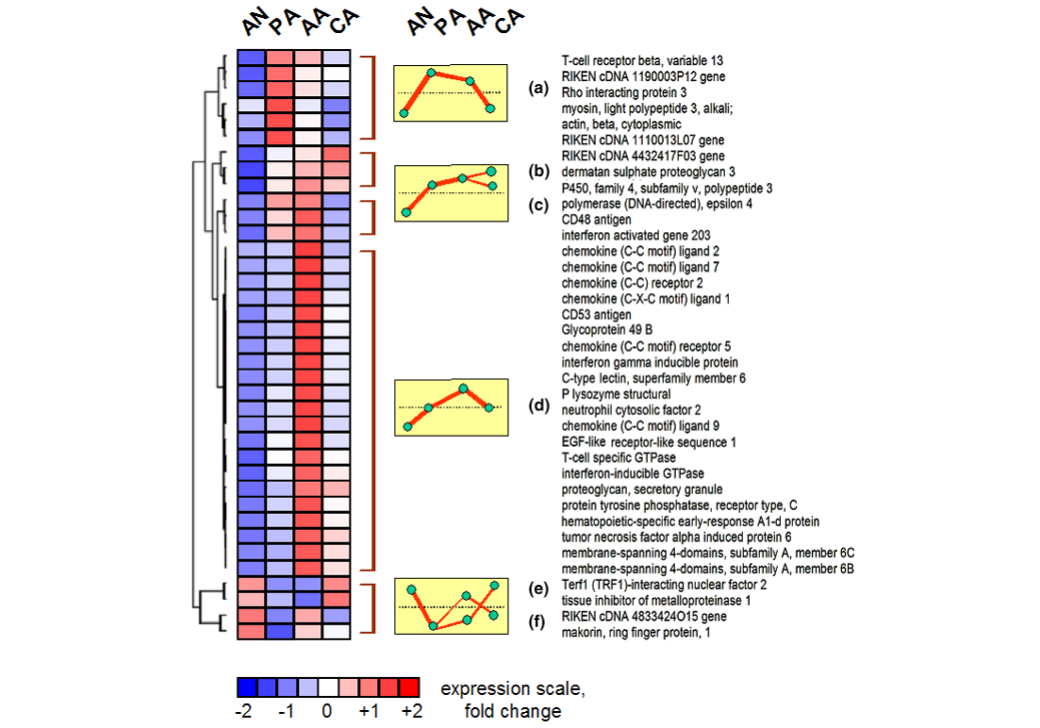
Hierarchical clustering (left) and expression patterns (A–F) for 37 early arthritis genes (listed in Table 2) differentially expressed in pre-inflamed (PA) joints of recipient BALB/c^SCID ^mice. Gene expression was compared with normal paws (AN) of naive BALB/c^SCID ^mice (PA/AN comparison, with a cut-off threshold at twofold change). The expression profiles of these 37 signature genes are shown for each phase of the disease (PA, acute [AA], or chronic [CA]) and also in normal paws.

Hierarchical clustering of a large number of phase-specific genes (*n *= 507) (Fig. 4) obscured the expression pattern of a relatively small number (*n *= 37) of early arthritis genes (Table 2). A separate hierarchical clustering was therefore performed for these 37 early genes, and the levels of expression were monitored at later phases of the disease. Six distinct expression patterns were identified (Fig. 5, clusters A–F) using this approach. Clusters A–D contained early arthritis genes whose transcription increased as the disease progressed, reaching a peak in the pre-inflamed joint or during inflammation. Cluster A included genes that coded for variable parts of the T cell receptor, together with genes related to cytoskeleton reorganization such as Rho interacting protein 3, myosin, and β-actin (reviewed in [52,53]). Cluster A genes were at the peak of their expression in the PA joint. However, most early arthritis genes in clusters C and D showed an expression peak later, at the acute phase of inflammation (Fig. 5), and encoded chemokine receptors (*Ccr2 *and *Ccr5*) and chemokine ligands (*Cxcl1*, *Ccl2*, *Ccl7*, and *Ccl9*). Clusters C and D also included interferon-activating genes *Ifi203*, *Ifi47*, and *Ifigtp*, and cell differentiation antigens such as CD48 and CD53.

Hierarchical clusters E and F contained four genes whose expression was downregulated in the pre-inflamed joint but returned to a 'normal' level (as expressed in naive paws) during arthritis progression. Clusters E and F included genes encoding Terf1-interacting nuclear factor 2, tissue inhibitor of metalloproteinase 1, makorin, and DNA clone *4833424O15 *with unknown function (Table 2 and Fig. 5).

## Discussion

This study describes genome-wide gene activity taking place in mouse joints during three major phases of autoimmune arthritis: initiation, acute inflammation, and chronic inflammation. Spleen cells from PG-immunized arthritic BALB/c mice were used to transfer the disease into non-immunized syngeneic SCID mice [30,32]. This adoptive transfer system minimized the individual differences that are typical in primary arthritis (induced by systemic immunization), and also excluded antigen-independent stimulation of the immune system by the adjuvant. Additional benefits of the cell transfer included a decrease in the time needed for arthritis development, and uniformity and synchronization of joint inflammation in recipient mice [32].

Two major criteria were used to select genes that might be important for arthritis development: (1) significant differences in expression levels between experimental groups and (2) the fold change in expression levels. When only the first criterion was applied, genome-wide analysis identified a large number of genes whose expression was significantly (*P *< 0.05) different between any pair of the experimental conditions compared. Irrespective of the statistics used (either unpaired Student's *t*-test, the Fisher exact test or the Mann–Whitney *U*-test), the number of differentially expressed genes was found to represent about 5–10% of the entire mouse genome. We further 'filtered' these genes by using a cut-off threshold set at twofold change of expression, because this threshold could reflect a physiologically important change in gene activity, and a twofold change exceeded the average CV for all pairwise comparisons. Decreasing the number of 'false positive' genes by application of these two filtering procedures proved to be an effective technique for the identification of genes that are likely to be involved in arthritis development.

The present study indicates that the number of differentially expressed genes increases with the progression of the disease. At the initiation phase, when no clinical symptoms of inflammation were yet detected, only 37 genes were upregulated or downregulated. However, a differential expression of 277 genes was observed at the acute phase, and chronic inflammation was characterized by the differential activity of 418 genes. Interestingly, most early arthritis signature genes (27 of 37) remained upregulated or downregulated in inflamed joints (Fig. 2). A different set of genes was also involved in acute inflammation. At the chronic phase, less than half of AA-specific genes (127 of 277) were differentially expressed, and another half was CA-specific. A very limited number of transcripts (*n *= 15) remained upregulated or downregulated in all three phases of arthritis.

Activated T cells must be present in the peripheral blood of recipient BALB/c^SCID ^mice after the transfer, but donor lymphocytes can be detected in joints as early as 3–5 days after the second transfer [32]. In earlier studies [31], and in control experiments (data not shown), using fluorescein-labeled or isotope-labeled donor lymphocytes, only very few cells were found in joints during the first week of transfer, and a second cell transfer was needed to induce a significant influx of lymphocytes into the joints and cause subsequent inflammation. In this study, we detected overexpression of a T cell-specific GTPase (*Tgtp*) and T cell receptor β (*Tcrbv13*) in still non-inflamed (pre-arthritic) paws of recipient BALB/c^SCID ^mice as early as 3–5 days after the second injection, indicating the presence of donor BALB/c lymphocytes. Thus, the initiation and development of arthritis in adoptively transferred PGIA must depend on cooperation between adaptive immunity cells (represented by donor BALB/c lymphocytes) and cells of innate immunity (represented by non-lymphoid cells in the recipient BALB/c^SCID ^mice). Analysis of the cellular and tissue specificity of gene expression, using public gene expression databases [54-56], indicated that genes encoding CD48 (*Cd48*), membrane-spanning 4A6B and 4A6C (*Ms4a6b *and *Ms4a6c*), epidermal growth factor-like receptor-like protein 1 (*Emr1*), and interferon-induced 47 kDa protein (*Ifi47*) were most probably originating from donor lymphoid cells, whereas other early arthritis genes (Table 2) were related to the activation of the innate immune system (represented by macrophages, dendritic cells, and cells of myeloid lineage) of recipient BALB/c^SCID ^mice.

Transcriptional control of gene activity is only one component of the complex cellular regulatory pathways. In other words, the functional activity of a protein depends on several factors such as interaction with other proteins, phosphorylation/dephosphorylation, subcellular compartmentalization, and other post-translational modifications. All of these factors might be involved in the regulation of interactions between the donor lymphocytes and the synovial/joint cells of recipient mice that lack an adaptive immune system. The list of genes we present in this study is rather short; that is, it includes only genes profoundly affected during arthritis initiation and progression at the level of transcription. Genes and proteins that are under subtle regulatory pressure, or are controlled by non-genetic mechanisms such as protein phosphorylation and other post-translational events, could not be detected and analyzed in this study. The development of new proteomics assays, and the synthesis of existing knowledge in cellular signaling pathways with information provided by gene expression studies, will be necessary to build up a complete arthritis-related regulatory network and to unravel the mechanisms involved in the development and progression of autoimmune arthritis.

## Conclusions

The development and progression of a complex polygenic autoimmune disease such as RA are controlled by hundreds or thousands of genes, in addition to the MHC. Despite the relatively high incidence of RA in the human population, only a few studies have applied gene array methods to the monitoring of disease progression and efficacy of treatment, or to predicting the prognosis of the disease. The major obstacles in the human studies are the relatively late diagnosis of RA, the large variety of cell types (cells of the immune system and of synovial joints) involved in autoimmune arthritic processes, and the extreme genetic heterogeneity of the human population. The present study applied an adoptively transferred murine model of RA and a microarray approach to detect differentially expressed, disease-related signature genes in PA (still non-inflamed) joints, days before the clinical symptoms or histopathological abnormalities of joint inflammation could be observed.

However, the detection of early arthritis signature genes in joints can be done only in an experimental system in which particular joints have already been affected before the inflammatory symptoms can be identified. To make this experimental system uniform, that is, to exclude individual variations, we adoptively transferred antigen (PG)-specific lymphocytes (representing cells of adaptive immunity) from primarily arthritic mice into syngeneic SCID mice, which lack an adaptive immune system. In this highly synchronized and uniform system we were able to detect differentially expressed genes in still non-inflamed paws of arthritis-'prone' animals. We identified a relatively small number of mostly upregulated early arthritis signature genes (known to be involved in arthritic processes and/or autoimmunity), some of which were expressed at even higher levels in the acute phase of arthritis. These early arthritis signature genes, originating from donor cells, indicated the involvement of adaptive immunity, whereas the innate immunity genes were differentially expressed by cells of the recipients.

The early signature genes, together with those that were differentially expressed in the acute (277 genes) and chronic (418 genes) phase of arthritis, are listed in the Additional files. Although many of these differentially expressed genes, detected either in the acute phase or during the progression of the disease, have been implicated in inflammation or autoimmunity, the list contains a significant number of differentially expressed genes whose function, or association with arthritis, is unknown at present.

## Abbreviations

AA = acutely arthritic; AN = absolutely negative (control naive); CA = chronically arthritic; CV = coefficient of variation; DDA = dimethyldioctadecylammonium bromide; PA = pre-arthritic; PG = cartilage proteoglycan aggrecan; PGIA = PG-induced arthritis; RA = rheumatoid arthritis; SCID = severe combined immunodeficient.

## Competing interests

The author(s) declare that they have no competing interests.

## Authors' contributions

VAA performed essentially all statistical analyses and put together the draft version of the results and figures. CV isolated all RNA samples, prepared biotinylated samples and was involved in Affymetrix hybridization experiments; he also performed preliminary clustering experiments with GeneSpring version 6.2 (not included in this paper). AH performed all *in vitro *stimulation and adoptive transfer experiments, and assessed arthritis three or four times a day together with KM, who was also involved in all phases of the experimental processes and in the finalization of the manuscript. EGB controlled Affymetrix hybridization and scanning experiments, managed preliminary data analysis and finalized the manuscript. TTG designed experiments, controlled all experimental steps, data analysis, and finalized the manuscript. All authors read and approved the final manuscript.

## Supplementary Material

Additional File 1 A table (Excel file) that lists all information about the 37 genes differentially expressed (more than twofold level) in pre-arthritic joints/paws, when compared with the same genes expressed in normal (naive) joints/paws of BALB/c^SCID ^mice (PA/AN comparison). The pre-arthritic (still non-inflamed) joints were collected within 24–48 hours after the onset of inflammatory symptoms in BALB/c^SCID ^mice with adoptively transferred PGIA. Acutely inflamed paws from these arthritic BALB/c^SCID ^mice were used as acute arthritic (AA) samples (the list of gene expression profiles is provided in Additional file 2.) Click here for file

Additional File 2 This file contains information about 256 upregulated and 21 downregulated genes in acutely arthritic joints/paws in five independent hybridization experiments. All genes with twofold or higher expression levels are listed. Click here for file

Additional File 3 This file includes 201 upregulated and 217 downregulated genes in subacute/chronic phase of arthritis (8–12 days after onset) with the corresponding information. The gene expression levels (twofold or higher) are compared with those expressed in normal joints of naive (absolutely negative) BALB/c^SCID ^mice. Click here for file

## Figures and Tables

**Table 1:** Experimental groups used for adoptive disease transfer and differential expression analysis

Group	RNA source	Treatment	Days after injection	No. of animals
AN	Naive control (absolute negative) BALB/c^SCID ^paw	None	N/A	3
PA	Normal paw from arthritic BALB/c^SCID ^mouse	Cell transfer	6	3
AA	Acute arthritic paw of BALB/c^SCID ^mouse	Cell transfer	6	5
CA	Chronically arthritic paw of BALB/c^SCID ^mouse	Cell transfer	12–14	3

Group AN represents naive BALB/c^SCID ^mice that received no cells. Experimental groups PA, AA, and CA received antigen-stimulated lymphocytes from arthritic BALB/c donor mice. RNA was isolated from four paws of two mice at the indicated number of days after injection, and pooled.

**Table 2:** Array-based expression values of upregulated or downregulated genes in pre-inflamed joint

Affy ID	Description	Gene	Mean AN expression	AN presence call	Mean PA expression	PA presence call	Fold	Cluster
161968_f_at	**Chemokine (CC motif) receptor 5**	*Ccr5*	1	A	57.1	P	57.1	D
95349_g_at	Chemokine (CXC motif) ligand 1	*Cxcl1*	1	A	55.9	P	55.9	D
104750_at	**Interferon-γ inducible protein**	*Ifi47*	0.69	A	15.69	P	22.7	D
130509_at	Membrane-spanning 4-domains member A6C	*Ms4a6c*	1.17	A	10.27	P	8.78	D
93106_i_at	T-cell receptor beta, variable 13	*Tcrbv13*	1.86	A	10.34	P	5.55	A
98474_r_at	Tumor necrosis factor-α induced protein 6	*Tnfaip6*	3.61	A	19.97	P	5.54	D
94761_at	**Chemokine (CC motif) ligand 7**	*Ccl7*	24.47	A	115.33	P	4.71	D
101578_f_at	Actin, β, cytoplasmic	*Actg*	188.33	P	859.16	P	4.56	A
102736_at	**Chemokine (CC motif) ligand 2**	*Ccl2*	29.89	P	125.48	P	4.20	D
95121_at	Polymerase (DNA-directed) ε 4 p12	*Pole4*	8.95	P	26.13	P	2.92	C
93397_at	**Chemokine (CC) receptor 2**	*Ccr2*	93.67	P	259.21	P	2.77	D
102906_at	**T cell-specific GTPase**	*Tgtp*	24.27	A	66.03	P	2.72	D
97322_at	Membrane-spanning 4-domains member A6B	*Ms4a6b*	9.43	A	25.16	P	2.67	D
93514_at	Myosin, light polypeptide 3	*Myl3*	29.28	A	77.42	P	2.64	A
103089_at	CD48 antigen	*Cd48*	34.17	A	85.82	P	2.51	C
103507_at	**EGF-like hormone receptor-like sequence 1**	*Emr1*	59.04	A	145.95	P	2.47	D
96764_at	**Interferon-inducible GTPase**	*Ifigtp*	66.38	A	157.88	P	2.38	D
102326_at	Neutrophil cytosolic factor 2	*Ncf2*	23.67	P	55.88	P	2.36	D
104388_at	**Chemokine (CC motif) ligand 9**	*Ccl9*	246.56	P	568.47	P	2.31	D
93321_at	Interferon-activated gene 203	*Ifi203*	43.58	P	99.27	P	2.28	C
94085_at	Proteoglycan, secretory granule	*Prg*	329.68	P	747.2	P	2.27	D
92762_at	**C-type lectin, superfamily member 6**	*Clecsf6*	35.73	A	80.64	P	2.26	D
93136_at	Dermatan sulphate proteoglycan 3	*Dspg3*	30.23	A	67.77	P	2.24	B
101753_s_at	P lysozyme structural	*Lzps*	635.78	P	1398.72	P	2.20	D
94939_at	CD53 antigen	*Cd53*	112.16	P	243.17	P	2.17	D
94958_at	RIKEN cDNA 1110013L07 gene	*1110013L07Rik*	24.91	A	53.7	P	2.16	A
162066_f_at	Rho interacting protein 3	*Rip3*	19.15	A	40.54	P	2.12	A
93039_at	RIKEN cDNA 1190003P12 gene	*1190003P12Rik*	36.44	P	77.03	P	2.11	A
101048_at	Protein tyrosine phosphatase, receptor type, C	*Ptprc*	138.7	A	289.19	P	2.09	D
92217_s_at	Glycoprotein 49 B	*Gp49b*	72.11	P	148.01	P	2.05	D
93869_s_at	**Hematopoietic-specific A1-d protein**	*Bcl2a1a*	49.43	A	100.11	P	2.03	D
103989_at	RIKEN cDNA 4432417F03 gene	*4432417F03Rik*	35.66	A	72.18	P	2.02	B
160611_at	**Cytochrome P450 polypeptide 4v3**	*Cyp4v3*	82	P	164.59	P	2.01	B
162107_r_at	**Tissue inhibitor of metalloproteinase 1**	*Timp1*	9.64	P	4.39	A	-2.20	E
164493_i_at	**Makorin, ring finger protein, 1**	*Mkrn1*	8.23	P	3.21	A	-2.56	F
167637_i_at	RIKEN cDNA 4833424O15 gene	*4833424O15Rik*	8.19	P	2.64	A	-3.11	F
167950_r_at	Terf1 (TRF1)-interacting nuclear factor 2	*Tinf2*	8.82	P	1.65	P	-5.35	E

Affy ID, unique Affymetrix probe set identifier. Description, gene description. Gene, gene abbreviation. Mean AN expression, average expression value in basal experimental condition of clinically normal paws of naive severe combined immunodeficient mice without cell transfer. Mean PA expression, average expression value in pre-arthritic joints. Presence call, average presence call for gene in AN or PA experimental condition: P, transcript was actually present in the majority of samples; A, transcript was actually absent in the majority of samples. Fold, fold change in gene expression in PA joint compared with AN basal expression. Cluster, cluster designation from Fig. 5. Difference in expression was significant by Mann–Whitney *U*-test, *P *< 0.05. Differential expression for listed genes was either greater than twofold overexpression or less than twofold downregulation (negative values). Genes that were differentially expressed in both pre-inflamed paws and *in vitro*-stimulated lymphocytes used for cell transfer are shown in bold type.
